# Impact of hydatid cyst laminated layer antigens on cell death rate, apoptosis induction, and key genes in the cell proliferation pathway: Insights from A549 cell line studies

**DOI:** 10.1371/journal.pone.0335188

**Published:** 2025-10-24

**Authors:** ElaheSadat Hosseini, Masoumeh Tavakoli-Yaraki, Ahmad Reza Meamar, Zeynab Ajam, Maryam Alipour, Ruchika Bagga, Elham Razmjou, Raheleh Rafiei-Sefiddashti

**Affiliations:** 1 Department of Parasitology and Mycology, School of Medicine, Iran University of Medical Sciences, Tehran, Iran; 2 Department of Clinical Biochemistry, School of Medicine, Iran University of Medical Sciences, Tehran, Iran; 3 Fortis Memorial Research Institute, Gurgaon, Haryana, India; Northwest University, UNITED STATES OF AMERICA

## Abstract

While lung cancer remains a lethal disease despite treatment advances, some parasitic infections can demonstrate cancer-modulating roles and exhibit anti-tumor effects. The emergence of hydatid cysts as a potential anti-cancer treatment has sparked optimism for the development of more successful therapies. This research examines the effect of hydatic cysts on the growth and proliferation of lung tumor cells, as well as the underlying molecular mechanisms involved. The laminated layer (LL) of the hydatid cyst antigens was administered to lung cancer cells with varying dosages and durations. The MTT assay was applied to evaluate cell viability. After exposure to different concentrations of LL antigens, the apoptosis, necrosis, cell cycle, and intracellular reactive oxygen species (ROS) of the cell culture were measured using flow cytometry. The expression levels of SOX-9, β-catenin, CD133, and CD44 genes were assessed using Real-Time PCR. Treating A549 cells with varying concentrations of LL antigens resulted in a decrease in viable cells, which depended on both time and dosage. Treatment with cysts led to apoptosis induction and a reduction in necrosis percentage in a dose-dependent manner. The induction of apoptosis correlated with elevated ROS production and a notable decrease in the expression of invasion-related genes (β-catenin, CD133, and CD44) (P < 0.05). However, this reduction in expression was not statistically significant for SOX-9. Exposing lung cancer cells to precise amounts of crude LL antigens resulted in cell death, apoptosis, increased intracellular ROS levels, and reduced expression of genes linked to cancer cell growth and invasion. These results lay the groundwork for further exploring purified *Echinococcus granulosus* parasite antigens as potential drug targets in cancer treatment.

## Introduction

Cancer is a significant global threat to human life. Annually, approximately 20 million new cancer cases and nearly 10 million deaths are reported worldwide [1]. Among these, lung cancer stands out as one of the most prevalent malignancies [2]. Lung cancer is a complex disease capable of metastasizing locally or even throughout the body [3,4]. The standard treatments involving surgery, radiotherapy, and chemotherapy, challenges such as tumor cell metastasis, harm to healthy cells, suppression of the immune system, drug toxicity, and drug resistance persist. Therefore, extensive efforts are being made to discover suitable therapeutic alternatives. The ancient idea that various biological agents, including bacteria, yeasts, viruses, and parasites, can be used as cancer treatments is gradually gaining attention. The positive impacts of certain parasitic infections on tumors include triggering apoptosis, boosting the immune system, preventing metastasis, and controlling angiogenesis signals to control cancer development [5].

Hydatid disease (Cystic Echinococcosis or CE) is caused by the larval stage of *Echinococcus granulosus* in the liver and lungs as primary infection sites [6]. Interestingly, research has demonstrated that individuals with CE may have a lower incidence of cancer compared to those without such cysts, suggesting that an *E. granulosus* infection may serve as a protective factor against cancer [7–11]. A study of 3,300 hepatic CE patients and 815 hepatocellular carcinoma (HCC) patients found only 13 cases of co-occurrence (0.39% incidence), significantly lower than expected. The CE + HCC cohort had prolonged median survival, suggesting CE may delay HCC progression [12]. A study of 2,086 solid tumor patients (1990−2001) found only 2 cases of concurrent hydatid disease, showing extremely rare co-occurrence (0.096% prevalence) [13]. These cysts are a fluid-filled structure comprising three layers: the adventitial layer, formed by host-parasite reaction; the laminated layer; and the inner layer called the germinal layer [14]. *E. granulosus* might decrease cancer risk by secreting molecules that can be developed as anti-cancer therapeutic drugs [15,16]. It can also induce this effect in the murine model, presumably through activating Th-1-polarized immune response with common antigens, especially the mucin-type O-glycans, and secreting molecules with anti-cancer potential, EgKI-1 in particular [16,17].

Analyzing the gene profile of β-catenin and CD133 could indicate the epithelial-mesenchymal transition (EMT) process under the induced conditions in the cells, which play a role in tumor progression and resistance to treatment [18]. CD133, a membrane glycoprotein involved in cell membrane organization, is overexpressed in tumor tissue and is regarded as a diagnostic indicator [19]. SOX-9, as a transcription factor, plays the role of a proto-oncogene and can influence the microenvironment of the tumor [20]. Beta-catenin plays an essential role in the proliferation of cancer cells and their invasion [21]. Furthermore, the increased expression of CD44 aids in promoting the migration of tumor cells [22].

This study aimed to investigate the effects of hydatid cyst antigens on cell proliferation, cell death, and invasion in vitro. Additionally, the study examines how the expression levels of SOX-9, β-catenin, CD133, and CD44 genes change in human lung adenocarcinoma (A549) cells after treatment with parasitic antigens.

## Materials and methods

### Ethical approval and consent to participate

The protocols for this study were reviewed by the Ethics Committee of Iran University of Medical Sciences and approved on December 29, 2021, under the code IR.IUMS.FMD.REC. 1400.584.

### Samples collection and preparation of the crude extract of LL

Sheep lung and liver samples naturally infected with hydatid cysts were collected post-slaughter (following Islamic protocols) from Karaj slaughterhouses (Alborz, Iran), where such contaminated tissues are routinely discarded as waste, requiring no special permissions for research use, and were subsequently transferred to our laboratory for analysis. The hydatid fluid was aspirated, and after observation of the protoscolices, the laminated layer)LL (was separated and washed with PBS [23,24]. The crude extract of the hydatid laminated layer was prepared according to the previous protocol [25,26]. LL was cut into small pieces, homogenized, and sonicated for 20 min in PBS buffer containing 1% penicillin-streptomycin (1M) on ice, centrifuged at 4°C, 2000 × g for 20 min. The supernatant protein concentration was measured using the Bradford method and then stored as the crude extract antigens of LL at −80°C before use [11,27–29].

### Cell culture

The A549 cell line, derived from adenocarcinomic human alveolar basal epithelial cells obtained from the Pasteur Institute of Iran, was cultured in Dulbecco’s Modified Eagle Medium (DMEM) high Glucose supplemented with 10% fetal bovine serum (FBS) and 1% penicillin-streptomycin in a humidified atmosphere of 5% CO_2_ at 37°C. When the cells reached 70–90% confluence, they were detached using trypsin-EDTA and subcultured. The cells were fresh, and their stocks were frozen at −80°C for further experiments.

### Cell viability assay (MTT assay)

The cytotoxicity of LL crude antigens on A549 cells was examined using the MTT assay. Briefly, A549 cells were seeded in DMEM in 96-well plates at a 9 × 10^3^ cells/well and allowed to adhere for 24 h at 37°C and 5% CO_2_. While cells reached sufficient confluence, they were treated with different dosages of LL crude antigens (50, 100, 200, 300, 400, and 500 µg/ml of LL) and incubated at 37°C for 24 and 48 hours [30]. After the incubation period, 20 µL of MTT solution at a concentration of 5 mg/mL in PBS was added to each well and incubated for 4 hours at 37°C. Subsequently, the wells’ supernatant was removed, and the formazan crystals were dissolved in 200 µL of dimethyl sulfoxide (DMSO). The absorbance values were then measured at 570 nm using a microplate reader from Tecan, Austria. The percentage of cell viability was determined by comparing the absorbance values of the treated and control groups [31]. Traditional IC50 determination may not fully apply to LL antigens, as these complex biological mixtures exhibit pleiotropic effects that complicate classical dose-response interpretations.

### Apoptosis assay

The apoptotic cell’s cytoplasmic membrane structure can change by exposing phosphatidylserine on its surface, a characteristic that can be identified using annexin V [32]. To detect apoptosis, 6 × 10^5^ A549 cells were seeded in 6-well plates containing DMEM supplemented with 10% FBS, 1% penicillin-streptomycin, and incubated. After a 24-hour incubation period, the cells were treated with varying concentrations of LL antigens and further incubated at 37°C for 24 hours. Subsequently, the cells were washed with PBS, resuspended in 500 µL of binding buffer, and exposed to 5 µL of Annexin V-FITC and 5 µL of propidium iodide (PI) before being incubated for 10 minutes at room temperature. The percentage of apoptotic cells was quantified and analyzed using a FACSCalibur flow cytometer (Becton Dickinson, San Jose, USA) along with its accompanying software (Cell Quest software) [33,34].

The flow cytometry data were analyzed with FlowJo software. Cell populations were gated using forward and side scatter parameters to remove debris, and then the fluorescence intensity of the specific markers was analyzed.

### Cell cycle analysis

The cell cycle distribution was determined using a Cell Cycle Detection Kit and analyzed with a FACSCalibur flow cytometer from Becton Dickinson in San Jose, USA, along with the BD Cell Quest software. In summary, cells were exposed to various concentrations of LL antigens and incubated at 37°C for 24 hours. Subsequently, DNA-binding dyes (such as propidium iodide or PI/RNase) were introduced into each well. It is important to note that ribonuclease was applied to the cells to ensure that only DNA, not RNA, was stained. The forward scatter (FS) and side scatter (SS) were measured to identify the cells in different phases of the cell cycle [35].

### Measurement of intracellular reactive oxygen species (ROS)

To measure the production of intracellular reactive oxygen species (ROS), a DCFH-DA Probe-Based ROS Detection Kit was utilized, and analysis was conducted using the FACSCalibur flow cytometer from Becton Dickinson in San Jose, USA. The cells were treated as described in the previous section and incubated with DCFH-DA and PBS for 20 minutes. Subsequently, the concentration of ROS within the cells was detected and analyzed using the flow cytometer [36,37].

### Gene expression analysis (SOX-9, β-catenin, CD133 and CD44)

**Fig 1:**
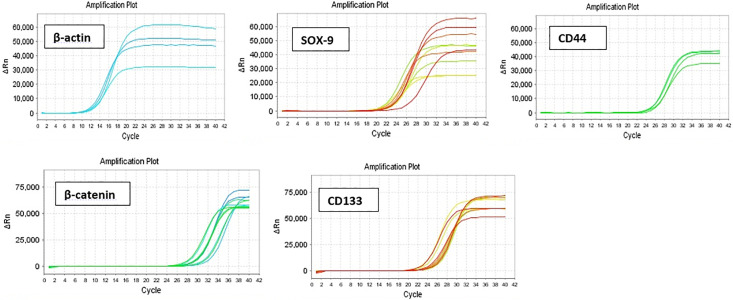
Amplification plot for β-actin, SOX-9, β-catenin, CD133, and CD44 expression. The treated and the control groups were statistically analyzed and compared using non-parametric one-way analysis of variance (ANOVA) with Dunnett’s post hoc test and Tukey’s post hoc test. The expression levels of selected genes of SOX-9, β-catenin, CD133, and CD44 between the tested groups were evaluated using the Kruskal-Wallis and Mann-Whitney U tests. All cell line experiments were conducted in triplicate and repeated three times to ensure specificity and accuracy. Graph Pad Prism version 9 (Graph Pad Software, San Diego, California) was utilized for all statistical calculations. The data were presented as mean±SD, and statistical significance was determined by asterisks denoting P < 0.05, P < 0.01, and P < 0.001 in the corresponding figures.

The SYBR-green-based quantitative reverse transcription polymerase chain reaction (RT-PCR) technique was applied to evaluate gene expression levels. Briefly, A549 cells (6 × 10^5^) were seeded in 6-well plates in DMEM supplemented with 10% FBS and 1% penicillin-streptomycin and incubated for 24 hours. Cells were treated with different concentrations of LL antigens, and following 24 hours of incubation, the genomic content (total RNA) was extracted from each well to measure the mRNA expression of the SOX-9, β-catenin, CD133, and CD44 using the Qiagen Kit, according to the manufacturer’s protocol [38]. Quantity, purity, and optimum concentration of harvested RNAs were measured with a nanodrop spectrophotometer (Thermo Scientific™ NanoDrop 2000c spectrophotometer) in a 260/280 nm ratio. Later, cDNA was synthesized using the RNA as a template [39,40]. RT-PCR was performed in a LightCycler 96 thermal cycler by RT-specific primers (Table 1) and Beta-actin, which was used as a reference gene to normalize the data [41]. The final volume for each reaction was a 20 µl mixture consisting of 10 µl of SYBR Green qPCR Master mix, 2 µl of cDNA, 1 µl of reverse primer, 1 µl of forward primer, and 6 µl of nuclease-free distilled water. In the amplification program, the first denaturation was carried out at 95°C for 10 min and continued with 40 cycles, consisting of a 95°C denaturation step for 10 sec, a 55°C annealing step for 20 sec, and a 60°C extension step for 35 sec (Fig 1). The comparative 2^-ΔΔCT^ method was applied for gene expression analysis [42].

**Table 1:** Primer sequence of SOX9, CD44, β-actin, CD133, and β-catenin of human genes.

Gene	Forward Primer 5′→3′	Reverse Primer 5′→3′	Tm
SOX9	CAACGGCTCCAGCAAGAACA	GCTTCTCGCTCTCGTTCAGA	58
CD44	GGAACAGTGGTTTGGCAACA	CTCTGCTGCGTTGTCATTGA	58
β-actin	TGG GCATCCACGAAACTAC	GATCTCCTTCTGCATCCTGT	57
CD133	AACGAACAGCATTTCTCTCTCAAGA	AACCTACAGCATATTCTTCA	58
β-catenin	ACTAGTCGTGGAATGGCACC	TGCAGTTCGCCTTCACTATG	57

## Results

1 **The effect of LL antigens on the cell growth inhibition, induction of apoptosis, the cell cycle progression, and ROS production of A549 cancer cell lines:**
  A **The effect of LL antigens on the **cell growth inhibition** of A459 cells**

**Fig 2:**
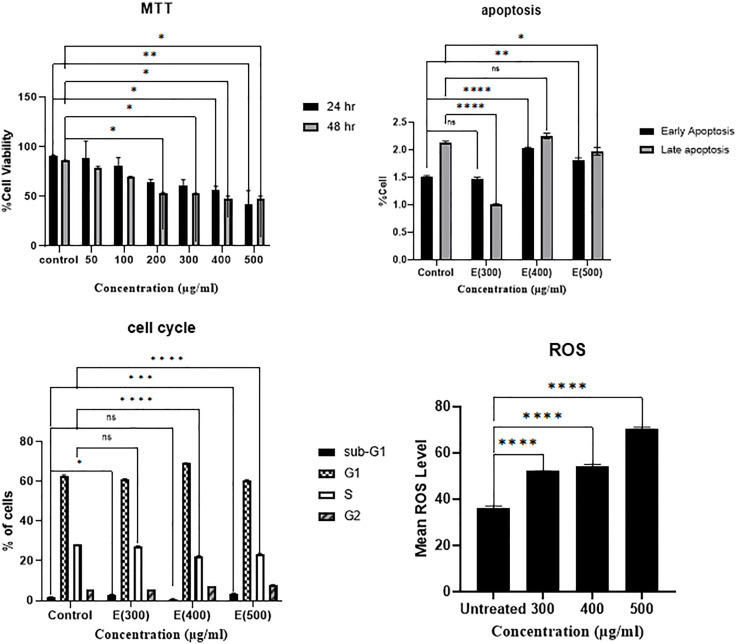
MTT assay, apoptosis, cell cycle, and ROS graphs. MTT assay graph, cells were treated with different concentrations (50, 100, 200, 300, 400, and 500 µg/ml) of LL antigens and incubated for 24 and 48 hours. Apoptosis, cell cycle, and ROS graphs, A549 cells were treated with 300, 400, and 500 µg/ml of LL and incubated for 24 hours. Statistical differences between treated and untreated control groups were analyzed by ANOVA (ns = P > 0.05, * = P < 0.05, ** = P < 0.01, *** = P < 0.001, and **** = P < 0.0001).

Based on data, a dose-dependent decrease in A549 cell viability was observed in both 24h and 48h (Fig 2). Treatment of A549 cells with 300, 400, and 500 µg/ml of LL reduced the cell viability by 60%, 56%, and 42% after 24 hours of incubation, respectively. According to the results, three concentrations of 300, 400, and 500 µg/ml following 24 hours of incubation were selected for further investigations on the possible induction of apoptosis, cell cycle distribution, ROS production, and gene expression.

B **The effect of LL antigens on the apoptosis of A459 cells**

Flow cytometry analysis demonstrated that treatment with hydatid cyst LL antigens increased the proportion of A549 cells undergoing early and late apoptosis in a dose-dependent manner. Specifically, the percentage of early apoptotic cells significantly increased with higher concentrations of LL antigens, reaching 400 µg/ml (P < 0.0001) and 500 µg/ml (P < 0.01) compared to untreated A549 cells (Fig 2). Flow cytometry histograms for each cell group, showing detailed percentages of viable cells (Annexin V − /PI−), early apoptotic cells (Annexin V + /PI−), and late apoptotic or necrotic cells (Annexin V + /PI+), are presented in S1 Fig. As shown, early apoptotic cells increased from 1.5% in control cells to 1.85% in cells treated with 500 µg/ml LL antigens. Conversely, necrotic cells decreased from 10.1% in control cells to 8.34% in the treated group, supporting the pro-apoptotic effect of LL antigens on lung cancer cells.

C **The effect of LL antigens on the cell cycle distribution of A459 cells**

The accumulation of cells in the sub-G1 phase of the cell cycle, which indicates cell cycle arrest, is a recognized hallmark of apoptosis. As shown in Fig 2, our results demonstrate that treatment with 500 µg/ml of LL antigen significantly increased the percentage of cells in the sub-G1 phase to 3.83%, compared to 0.85% in control cells (P < 0.0001). Additionally, there was a dose-dependent decrease in the percentage of cells in the S phase, declining from 30.97% in control cells to 23.83% following treatment with 500 µg/ml LL antigen. This reduction suggests a diminished proliferative capacity of cells after exposure to the LL antigen. The flow cytometry histograms depicting the cell cycle distribution are presented in S2 Fig.

D **The effect of LL antigens on the ROS level of A459 cells**

An increase in cellular ROS levels indicates cellular stress and is typically associated with the induction of apoptosis. In this study, the ROS levels in A549 cells were measured following treatment with escalating concentrations of LL antigens. As shown in Fig 2D, ROS levels increased significantly after treatment with 300 µg/ml (P < 0.0001), 400 µg/ml (P < 0.0001), and 500 µg/ml (P < 0.0001) of LL antigens compared to untreated cells. These results demonstrate the ability of LL antigens to induce cellular stress in A549 lung cancer cells. The flow cytometry histograms illustrating the ROS production patterns are presented in S2 Fig.

1. **The expression level of SOX-9, β-catenin, CD133, and CD44 in cells treated with LL antigens**

**Fig 3:**
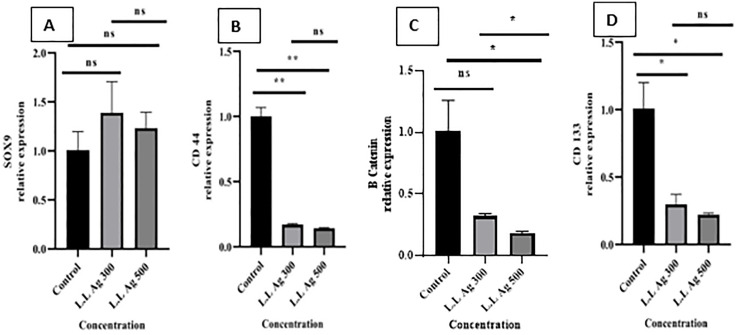
SOX-9, CD44, β-catenin, and CD133 gene expression graphs. SOX-9, CD44, β-catenin, and CD133 gene expression graphs in the sample of A549 cancer cells treated with antigens containing 300 and 500 μg/ml of protein concentration prepared from hydatid cyst layer laminate (L.L Ag) compared to A549 cancer cells not treated with this Antigen (control). (ns = P > 0.05, * = P < 0.05, ** = P < 0.01, *** = P < 0.001 and **** = P < 0.0001).

The SOX-9 expression level following exposure of cells to different concentrations of LL antigens showed no remarkable change compared to the untreated cells (Fig 3). The detected increase in the level of SOX-9 expression at the concentration of 300 µg/ml was not statistically significant (P = 0.2814).

As shown in Fig 3, the expression level of CD44 in the cells treated with 300 and 500 µg/ml of LL antigen was significantly decreased compared to the untreated cells (P-value = 0.0035 for 300 µg/ml and P-value = 0.0032 for 500 µg/ml). Also, the expression level of β-catenin in the treated cells decreased compared to the untreated cells. However, this decrease was only statistically significant in a concentration of 500 µg/ml (P = 0.0412 for the dose of 500 and P = 0.0578 for 300 µg/ml) (Fig 3). The expression of the CD133 gene in the treated cells significantly decreased compared to the untreated cells in both concentrations of 300 µg/ml (P = 0.039) and 500 µg/ml (P = 0.0284) (Fig 3).

## Discussion

Incident cases of cancer will increase by up to 64% by 2040, with global lung cancer deaths potentially reaching 3.2 million by 2050 [43–45]. Consequently, there is a push to discover effective treatments to decrease cancer rates and improve survival rates. Parasitic agents, including trematodes, have displayed promising antitumor properties [46]. For example, *E. granulosus* may inhibit neoplastic changes and suppress tumors [47,48]. This study was motivated by three key scientific imperatives. First, the impact of this parasite on lung cancer cell lines was studied outside of the body without interference from the host immune system. Second, we specifically investigated how laminated layer (LL) antigens may modulate cancer cell viability through ROS-mediated apoptosis, downregulate critical metastasis markers (β-catenin, CD133, CD44), and alter cell cycle dynamics in lung adenocarcinoma. Third, the dose/time-dependent responses observed (P < 0.05) establish proof-of-concept for parasite-derived molecules as potential adjuvants to existing therapies. This rationale bridges parasitology and oncology by demonstrating how evolutionary host-parasite interactions can yield unexpected therapeutic opportunities.

The levels of apoptotic and necrotic cells were evaluated to determine how LL antigens impact A549 cells. Apoptosis refers to a cellular self-destruction process that prevents inflammation. On the other hand, necrosis is characterized as a haphazard cell demise leading to the unregulated release of inflammatory cell components [49]. Notably, Gao et al. demonstrated that hydatid cyst fluid with a high concentration could be toxic to melanoma A375 cells, but these antigens in low dosage make progress in the cell cycle and increase the expression of antiapoptotic protein Bcl-2 [50]. In the Baysal study in 2021, the evaluation of the effect of hydatid cyst fluid on cell proliferation and the expression of some apoptotic genes in two cell lines, A549 (lung adenocarcinoma) and BEAS-2B (healthy lung epithelial), was investigated. Hydatid cyst fluid stopped cancer progression and prevented tumor formation. The results showed a statistically significant decrease in the expression of the BCL-2 gene and an increase in the expression of the P53 gene, which led to an increase in apoptosis [51]. Moreover, Díaz et al. demonstrated that the LL carbohydrates interact selectively with the Kupffer cell receptor. It was described that the LL molecular may also have immune-regulatory and anti-inflammatory properties [52–54]. Various parasites can disrupt the cell cycle and halt cell proliferation, underscoring the importance of evaluating the different phases of the cell cycle (G1, S, G2, and M phase) in cancer cells treated with parasite antigens [5]. Three synthetic small peptides derived from *E. granulosus* exhibited significant apoptosis and inhibited proliferation in HT29 and HepG2 cell lines by increasing the G0/G1 phase and decreasing the S phase [55].

ROS are crucial in various cellular processes, including gene expression and programmed cell death. In a study by Chunxue Fu et al. in 2024, it was demonstrated that the cystic fluid of *E. granulosus* elevated macrophage ROS levels, suppressing the interferon-I response [56]. Our research revealed that LL antigens can induce cell death in a dose- and time-dependent manner by promoting early apoptosis and impeding the cell cycle, mainly by reducing the S phase at 400 and 500 µg/ml concentrations. The escalation of intracellular ROS levels dose-dependently aligns with our hypothesis regarding the increased death of tumor cells. Therefore, it may be plausible to consider specific LL antigens as potential tumor biotherapy agents that drive cancer cells toward apoptosis through various mechanisms, including the augmentation of ROS levels.

Moreover, it was revealed that the levels of SOX9, β-catenin, CD133, and CD44 gene expressions have an impact on stem-cell properties, including self-renewal or EMT process, which can be the determining factor in the invasion and metastasis of tumor cells [57]. The increase in SOX9 levels enhances migration, invasion, and EMT through the Wnt/β-catenin pathway in lung cancer cell lines A549 [58]. In the current study, the expression levels of the SOX9 gene in the A549 cell line treated with LL antigens compared to untreated cancer cells were investigated. It can be postulated from our data that SOX-9 might have less contribution to LL antigen-induced cell death.

Parasites such as *Heligmosomoides polygyrus* can evade the host immune system by downregulating the production of TGF-β and suppressing CD44-T cells [59]. Also, some protozoa, like *Toxoplasma gondii* infection, could decrease CD44 expression [60]. On the other hand, the overexpression of CD44 has been detected in humans infected with the liver fluke *Opisthorchis viverrini*, which is associated with cholangiocarcinoma, and it was suggested that CD44v9 may be involved in the development of inflammation-associated cancer [61]. Taken together, it can be explained that down-regulating CD44 expression could further enhance the anti-cancer effect of LL antigens. This document could serve as a valuable resource in understanding and describing this parasite’s anti-cancer effect.

Specific agents like *T. gondii*, *Clonorchis sinensis*, and *Opisthorchis viverrini* can cause primary liver cancers by altering Wnt/β-catenin signaling. This alteration increases the human host’s infection and survival rates [62]. *S. mansoni* antigens can induce Wnt/β- catenin signaling, leading to colorectal carcinogenesis. On the other hand, certain helminth infections like *Heligmosomoides polygyrus* can reduce the severity and risk of developing cancer. It is suggested that this might occur by decreasing the adherens junction protein β-catenin expression on human colorectal cancer cell proliferation in vitro [63,64]. Our research supports this, showing that *E. granulosus* antigens can reduce the expression of the β-catenin gene, which can decrease the growth and metastasis of cancerous cells when treated with LL antigens.

It has been discovered that CD133 is linked to an increase in the formation of DNA lesions and DDR proteins in cholangiocarcinoma, which could lead to genetic instability and the development of cholangiocarcinoma with aggressive clinical features [65]. Moreover, CD133 can be used as a biomarker for the early detection of small-cell lung cancer [66]. It is hypothesized that the down-regulation of CD133 might mediate the deactivation of cancer stem cells and be applied to lung cancer therapy [67]. Interestingly, antigens found in the *E. granulosus* parasite have been shown to reduce the expression of the CD133 gene, which may lead to future biotherapy options for lung cancer treatment.

This study had some limitations. One significant challenge was the inability to access new tools for separating different proteins and compounds in their pure forms. Instead, crude antigens from one part of the parasite were used. Additionally, comparing non-cancerous cells alongside cancerous cells would have been beneficial. However, most studies use only cancerous cell lines to report the primary result of one experimental test. It is essential to recognize that the initial steps in cancer treatment often focus on cancer cells before considering their effects on normal cells. This approach is grounded in the understanding that therapies must first demonstrate efficacy against malignant cells, which can then be compared with their effects on non-cancerous tissues. For instance, once a treatment shows promise in targeting cancer cells effectively, subsequent studies often expand to include normal cell lines to evaluate selectivity and safety in a broader biological context [68]. While it is critical to assess the impact of LL antigens on healthy cells, the progression from testing on cancer cells to evaluating effects on normal cells is a standard practice in cancer research. Future studies should aim to provide comprehensive data comparing the effects of LL antigens on both cancerous and normal cells to fully address these important safety considerations.

Our study identifies significant changes in apoptosis and gene expression, but it does not thoroughly investigate the specific molecular pathways involved. Understanding these pathways is crucial for revealing the complex mechanisms behind the anti-cancer properties of biological agents. Future research should focus on characterizing distinct signaling pathways, such as the MAPK and PI3K/Akt pathways activated by ROS, which are important for mediating apoptosis and regulating invasion-related gene expression. Additionally, while our findings indicate a decrease in SOX-9, β-catenin, CD133, and CD44 expression, this may be linked to reduced activity of EMT-inducing transcription factors, suggesting a lower conversion of epithelial cells to a mesenchymal phenotype. A more comprehensive analysis of epithelial-mesenchymal transition (EMT) should include additional markers and signaling pathways, such as E-cadherin downregulation and the upregulation of N-cadherin and Vimentin, along with key transcription factors like Snail, Slug, ZEB, and TWIST. Finally, it should be mentioned that the current study had some limitations. To comprehensively evaluate the potential therapeutic specificity of hydatid cyst laminated layer antigens, it would be essential to extend the investigation to additional cancer cell lines from various tissue origins and relevant non-cancerous cell models. This approach would clarify whether the observed cytotoxicity and apoptosis induction are preferentially targeted toward cancer cells, thereby supporting the development of LL antigens as selective anti-cancer agents that future studies can warrant.

## Conclusions

Our in vitro findings suggest that laminated layer (LL) antigens in hydatid cysts show potential for treating lung cancer. Our study revealed that LL antigens can cause cell death in a dose- and time-dependent manner by promoting early apoptosis and hindering the cell cycle. These antigens may be used potentially as tumor biotherapy agents by driving cancer cells toward apoptosis through various mechanisms, including increasing levels of reactive oxygen species (ROS). The 400 and 500 µg/ml concentrations were particularly effective in reducing the S phase and increasing intracellular ROS levels. When lung cancer cells were treated with LL antigens, their growth was associated with a significant decrease in the expression of genes such as β-catenin, CD133, and CD44, which play crucial roles in cell proliferation and tumor growth. Any dysregulation or mutation in these genes can disrupt normal cellular processes, leading to uncontrolled cell growth, tumor formation, and metastasis. The decrease in the expression of these genes is believed to be caused by a reduction in the expression of epithelial-mesenchymal transition EMT-inducing transcription factors, which in turn reduces the transformation of epithelial cells into a mesenchymal phenotype. Or it can be stated that, activation of Wnt/β-catenin signaling by SOX9 promotes proliferation and inhibits apoptosis. CD44 and CD133 support stem cell characteristics and survival signaling, modulating cell cycle checkpoints and protecting cells from ROS-mediated damage. ROS can induce apoptosis, but cancer cells often upregulate antioxidant defenses through these pathways to maintain redox balance and avoid cell death. These findings could lay the groundwork for future research using purified *E. granulosus* parasite antigens in cancer treatment, and in vivo validation is needed to confirm these observations. Finally, it should be mentioned that the current study had some limitations. To comprehensively evaluate the potential therapeutic specificity of hydatid cyst laminated layer antigens, it would be essential to extend the investigation to additional cancer cell lines from various tissue origins and to relevant non-cancerous cell models. This approach would clarify whether the observed cytotoxicity and apoptosis induction are preferentially targeted toward cancer cells, thereby supporting the development of LL antigens as selective anti-cancer agents that can be warranted by future studies.

## Supporting information

S1 Fig The apoptotic effects of LL antigens on human lung cancer cells. The effects of LL antigens at various concentrations on the induction of apoptosis in A549 cells were determined by Annexin V-FITC and propidium iodide (PI) staining and flow cytometry. The flow cytometry histograms indicate (A): untreated control cells; (B): cells treated with 300 µg/ml; (C): cells treated with 400 µg/ml; (D): cells treated with 500 µg/ml of LL antigens following 24 hours of incubation. These plots display the distribution and percentages of viable cells (Annexin V − /PI−) (Q4), early apoptotic cells (Annexin V + /PI−) (Q3), late apoptotic or necrotic cells (Annexin V + /PI+) (Q2), and necrotic cells (Annexin V − /PI+) (Q1).
(JPG)

S2 Fig The effects of LL antigens on cell cycle distribution in human lung cancer cells. The cell cycle distribution was analyzed by flow cytometry using PI staining and the flow cytometry histograms are represented for (A): un-treated control cells; (B): cells treated with 300 µg/ml; (C): cells treated with 400 µg/ml; (D): cells treated with 500 µg/ml of LL antigens following 24 hours of incubation. The percentages of cells in each phase are indicated in a box within each histogram.
(JPG)

S3 Fig The effects of LL antigens on cellular ROS level in human lung cancer cells. The A549 cells were treated with increasing concentrations of LL antigens, separately, and subjected to ROS measurement using flow cytometry. (A) represents untreated control cells; (B) represents cells treated with 300 µg/ml; (C) represents cells treated with 400 µg/ml; (D) represents cells treated with 500 µg/ml of LL antigens following 24 hours of incubation. The mean DCFH values are indicated in each histogram for each group of cells.
(JPG)
