# Supplementary figures and images for: Impact of hydatid cyst laminated layer antigens on cell death rate, apoptosis induction, and key genes in the cell proliferation pathway: Insights from A549 cell line studies
Source: PLoS One. 2025 Oct 24;20(10):e0335188. doi: 10.1371/journal.pone.0335188 (PMC12551906; doi:10.1371/journal.pone.0335188)

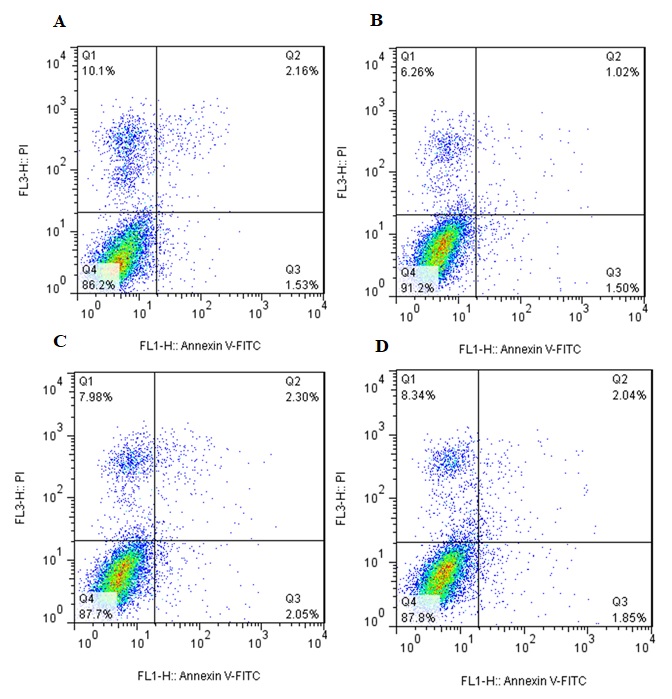

Supplement: S1 Fig — The effects of LL antigens at various concentrations on the induction of apoptosis in A549 cells were determined by Annexin V-FITC and propidium iodide (PI) staining and flow cytometry. The flow cytometry histograms indicate (A): untreated control cells; (B): cells treated with 300 µg/ml; (C): cells treated with 400 µg/ml; (D): cells treated with 500 µg/ml of LL antigens following 24 hours of incubation. These plots display the distribution and percentages of viable cells (Annexin V − /PI−) (Q4), early apoptotic cells (Annexin V + /PI−) (Q3), late apoptotic or necrotic cells (Annexin V + /PI+) (Q2), and necrotic cells (Annexin V − /PI+) (Q1). (JPG) [file pone.0335188.s001.jpg]

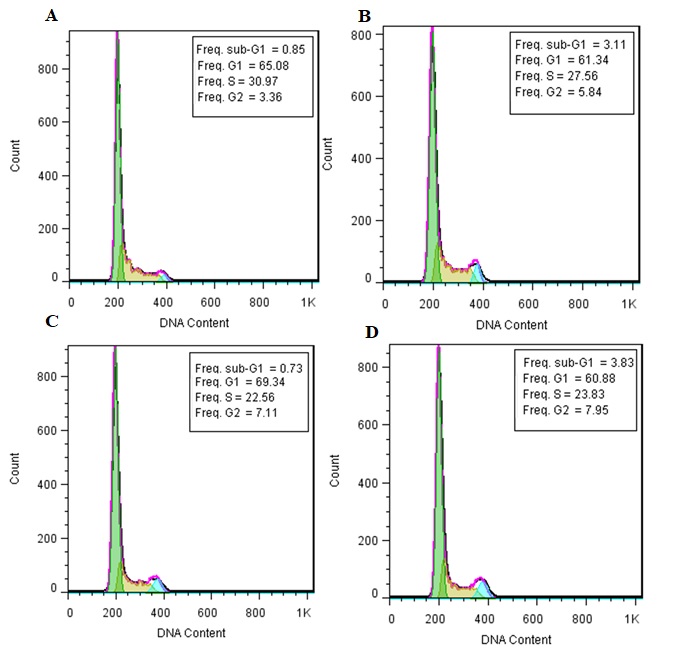

Supplement: S2 Fig — The cell cycle distribution was analyzed by flow cytometry using PI staining and the flow cytometry histograms are represented for (A): un-treated control cells; (B): cells treated with 300 µg/ml; (C): cells treated with 400 µg/ml; (D): cells treated with 500 µg/ml of LL antigens following 24 hours of incubation. The percentages of cells in each phase are indicated in a box within each histogram. (JPG) [file pone.0335188.s002.jpg]

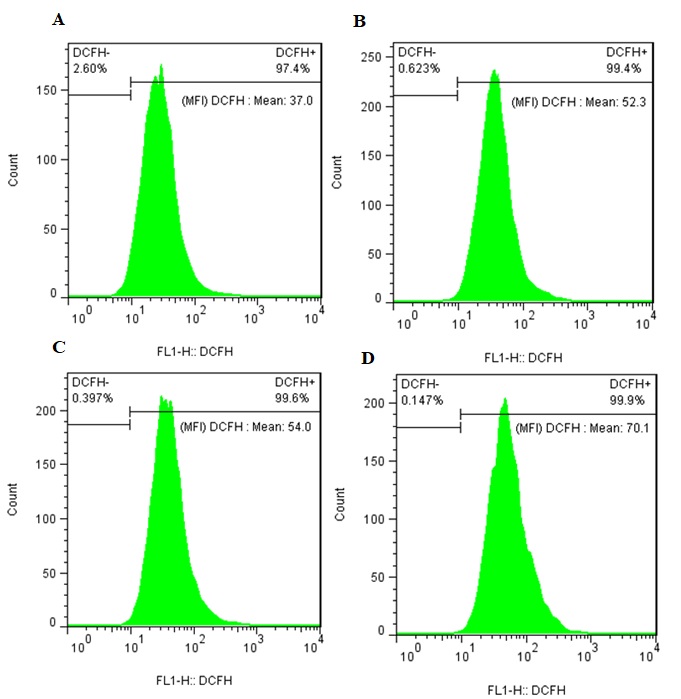

Supplement: S3 Fig — The A549 cells were treated with increasing concentrations of LL antigens, separately, and subjected to ROS measurement using flow cytometry. (A) represents untreated control cells; (B) represents cells treated with 300 µg/ml; (C) represents cells treated with 400 µg/ml; (D) represents cells treated with 500 µg/ml of LL antigens following 24 hours of incubation. The mean DCFH values are indicated in each histogram for each group of cells. (JPG) [file pone.0335188.s003.jpg]
